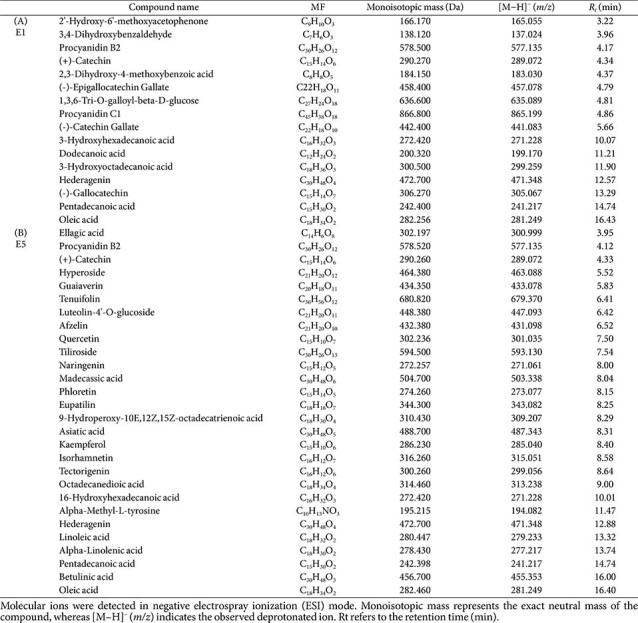# Anti-Acne Potential of *Quercus salicina* Extract: Inhibition of *Cutibacterium acnes* Growth and Virulence

**DOI:** 10.4014/jmb.2510.10052

**Published:** 2026-01-13

**Authors:** Suhyeon Hong, Sohae Park, Minkyoung Park, Jung Min Park, Dae Youn Hwang, Heeseob Lee, Jumin Park

**Affiliations:** 1 Department of Food Science and Nutrition, College of Human Ecology, Pusan National University, Busan 46241, Republic of Korea; 2 Department of Biomaterials Science (BK21 FOUR Program), Life and Industry Convergence Research Institute, College of Natural Resources and Life Science, Pusan National University, Miryang 50463, Republic of Korea

**Keywords:** *Quercus salicina*, *Cutibacterium acnes*, Antimicrobial, Virulence modulation

## Abstract

Acne vulgaris is a prevalent inflammatory skin disorder mediated by *Cutibacterium acnes*, a key etiological factor. In this study, the anti-acne properties of the ethyl acetate fraction of *Quercus salicina* Blume (QsB-EE) against *C. acnes* were investigated. The QsB-EE fraction was separated into subfractions (E1-E6) using preparative HPLC. Antimicrobial and anti-virulence activities were evaluated via bacterial growth, biofilm, and lipase activity assays. Virulence gene expression was assessed using qRT-PCR, and bioactive compounds were identified by LC-MS/MS. The QsB-EE demonstrated significant antimicrobial (MIC 125 μg/ml) and strong anti-virulence effects. The E1 fraction was the most potent, exhibiting the lowest MIC (16 μg/ml), highest biofilm inhibition (86.19% at 1,000 μg/ml), and highest lipase inhibition (93.72% at 10 μg/ml). In addition, mechanistic studies confirmed that E1 significantly downregulated the expression of key virulence genes: *ppa*0149 (biofilm), *geh*A (lipase), and *hyl* (hyaluronidase). LC-MS/MS identified (+)-catechin as the major compound in E1, alongside kaempferol and quercetin in E5. Although further *in vivo* studies are warranted to evaluate the therapeutic efficacy, our findings suggest that QsB-EE possesses promising anti-acne potential by targeting *C. acnes* virulence factors through the active compounds concentrated in the E1 fraction.

## Introduction

The gram-positive anaerobic bacterium *Cutibacterium acnes* is a key contributor to the pathogenesis of acne vulgaris, a common inflammatory skin disorder. This bacterium secretes virulence-associated enzymes, including lipase and protease, which trigger inflammation and induce skin cell damage [1]. Lipase, a key virulence factor, hydrolyzes triglycerides in the skin, leading to the release of free fatty acids that irritate the skin and exacerbate inflammation [2]. *C. acnes* also forms biofilms within the hair follicles, which further contribute to the pathogenesis of acne vulgaris. Biofilms enhance bacterial adhesion, increase resistance to antibiotic treatments, and promote the production of digestive enzymes [3]. The rising prevalence of antibiotic resistance in *C. acnes* underscores the urgent need for alternative antibacterial strategies. Inhibiting key virulence factors, particularly lipase and biofilm formation, is essential for effective acne management.

*Quercus salicina* Blume (QsB) is a species of oak belonging to the *Fagaceae* family, widely distributed in the southern regions of Korea, Taiwan, and Japan. In Korea and Japan, various *Quercus* species have a long history of use in traditional folk medicine for treating ailments such as diarrhea, dysentery, dermatitis, and hemorrhage [4]. QsB exhibits potent antioxidant activity and inhibits the skin enzymes tyrosinase and elastase, which suggests its potential for functional skincare applications [5]. Other studies have also reported the antioxidant, antibacterial, and enzyme inhibitory activities of various *Quercus* species [6]. QsB is particularly rich in phenolic compounds, surpassing the quantities present in other *Quercus* species [7]. These findings highlight the potential of QsB as a natural ingredient for pharmaceutical applications [8].

Currently, the main treatments for acne vulgaris include topical retinoids, antimicrobial agents, hormonal agents, and isotretinoin, but each has potential side effects [9]. Topical antibiotics like erythromycin and clindamycin may cause adverse effects, such as hyperkalemia, erythema, dryness and peeling, while extensive and irrational use can lead to antimicrobial resistance. Oral hormonal agents, although effective, can disrupt the endocrine system and cause side effects such as menstrual dysfunction [10]. Therefore, research is ongoing to develop biologically active compounds from natural substances with antibacterial properties comparable to conventional treatments, but without the associated side effects [11, 12]. Several studies have demonstrated the antimicrobial activities against *C. acnes* of various herbal extracts and essential oils, such as those of *Hemidesmus indicus*, *Eclipta alba*, *Ocimum basilicum* L. (sweet basil), and *Ocimum sanctum* L. (holy basil), thus confirming their potential for treating and preventing this disorder [13, 14].

In the present study, we investigated the antimicrobial potential of the ethyl acetate fraction of QsB (QsB-EE) against *C. acnes* and its mechanism of action. We evaluated its effects on bacterial growth, biofilm formation, and virulence-associated enzyme activity. Furthermore, the bioactive compounds responsible for these effects were identified using LC-MS/MS. These findings highlight QsB-EE as a promising natural alternative for acne treatment, with antimicrobial properties and better tolerability.

## Materials and Methods

### Chemicals and Reagents

Unless otherwise specified, all chemicals and reagents were of analytical grade. The 1,1-diphenyl-2-picrylhydrazyl (DPPH), 2,2-azino-bis(3-ethylbenzthiazoline-6-sulfonic acid) (ABTS), 4-methyl umbelliferyl oleate, caffeic acid, crystal violet, Folin-Ciocalteu's phenol reagent, naringin, and potassium persulfate were purchased from Sigma-Aldrich Co. (USA). Brain heart infusion broth (BHIB) was purchased from Difco Laboratories (USA). Ethyl acetate was obtained from Carlo Erba Co. (Italy). Acetic acid, agar powder, n-butanol, n-hexane, and methanol were obtained from Junsei Chemical Co. (Japan). Ethanol was purchased from Junsei Chemical Co. Diethylene glycol and methanol were obtained from Daejung Chemicals and Metals Co. (Republic of Korea). Dimethyl sulfoxide (DMSO) and sodium hydroxide solution (NaOH) were obtained from Duksan Pure Chemical Co. (Republic of Korea). Formic acid and the TOPreal SYBR Green qRT-PCR Kit were purchased from Thermo Fisher Scientific Inc. (USA) and Enzynomics (Republic of Korea), respectively. HPLC-grade acetonitrile (ACN), methanol, and water were purchased from Fisher Scientific Korea Ltd. Sodium citrate solution was purchased from Yakuri Pure Chemicals Co., Ltd. (Japan).

### Plant Material Collection and Preparation

The dried QsB material used in this study consisted of commercially supplied, dried whole leaves purchased from Jeju Island, Korea, in 2023. A voucher specimen (WPC-23-001) was deposited at the Functional Materials Bank (FMB) of the Pusan National University (PNU)–Wellbeing RIS Center. Upon receipt, the plant material was freeze-dried using a lyophilizer (Rikakikai Co., Ltd., Japan) and subsequently ground to a fine powder (100 mesh) using a grinder (Culatti AG, Switzerland). The resulting powder was stored at −20°C until use.

### Extraction and Fractionation of QsB

QsB powder (30 g) was extracted with 600 ml of 80% aqueous methanol (v/v) by shaking (200 rpm) in a shaking incubator (VS‐8480; Vision Scientific, Republic of Korea) at 25°C for 12 h. This extraction was repeated three times. The combined extracts were filtered through Whatman No. 2 filter paper and then concentrated under reduced pressure at 35°C using a rotary evaporator (EYELA, Japan). The concentrated extract was freeze-dried (FDU-2100, Rikakikai Co.) and stored at −20°C until use.

The 80% methanol extract (15 g) was dissolved in 300 ml of distilled water and sequentially partitioned (liquid-liquid extraction) with n-hexane, ethyl acetate (QsB-EE), and n-butanol. Each partitioning step was performed three times using a 1:1 ratio (v/v) of solvent to the aqueous solution, with shaking (200 rpm) at 25°C for 1 h per partition. Each resulting fraction was filtered, evaporated under reduced pressure, freeze-dried, and stored at −20°C.

### Antioxidant Assays

**DPPH radical scavenging assay.** DPPH radical scavenging activity was measured using a modified method of Blois (1958) and Baliyan *et al*. (2022) at 540 nm [15, 16]. A 0.06 mM DPPH solution was prepared in 95% ethanol and filtered through Whatman No. 2 filter paper. A 100 μl aliquot of the sample (dissolved in 50% ethanol) was mixed with 100 μl of the DPPH solution, followed by incubation for 30 min at room temperature in the dark. The absorbance was measured at 540 nm using a microplate reader (Sunrise, Tecan Co., Ltd., Switzerland) after brief shaking. The scavenging activity was expressed as a percentage of radical inhibition using the absorbance values of the control and samples. Ascorbic acid (100 μg/ml) was used as the positive control.

**ABTS radical scavenging assay.** ABTS radical scavenging assay was performed to measure the total antioxidant capacity, as previously described [17]. The ABTS radical cation solution was prepared by mixing 7 mM ABTS (in distilled water) with 2.45 mM potassium persulfate solution, followed by incubation for 12 h at room temperature in the dark. The solution was then diluted with 5 mM PBS (pH 7.4) until the absorbance at 734 nm reached 0.70 ± 0.02. Finally, 990 μl of the diluted ABTS solution was mixed with 10 μl of the sample, and the mixture was allowed to react for 6 min in the dark. The absorbance was subsequently measured at 734 nm using a spectrophotometer.

### Phytochemical Composition Analysis

**Determination of total phenolic content.** Total phenolic content (TPC) was determined using a modified Folin-Ciocalteu method [18]. Then, 50 μl of the sample was mixed with 500 μl of distilled water and 100 μl of Folin-Ciocalteu reagent. The mixture was incubated in the dark for 3 min. Subsequently, 100 μl of 10% Na_2_CO_3_ and 350 μl of distilled water were added, and the mixture was further incubated for 1 h at room temperature in the dark. After incubation, the absorbance was measured at 725 nm using a spectrophotometer. Caffeic acid was used as the standard, and the TPC was expressed as caffeic acid equivalents (CAE).

**Determination of total flavonoid content.** Total flavonoid content (TFC) was determined using a modified Davis method [19], whereby 100 μl of the sample was vortexed with 1 ml of 90% diethylene glycol solution. Subsequently, 100 μl of 1N NaOH was added, and the mixture was incubated for 1 h at 37°C. After incubation, the absorbance was measured at 420 nm using a microplate reader. Naringin was used as the standard, and the TFC was expressed as naringin equivalents (NE).

### Antimicrobial Activity Assays

**Bacterial strain and culture conditions.** The bacterial strain, *C. acnes* KCTC 3314, was purchased from the Korean Collection for Type Cultures (KCTC, Republic of Korea). *C. acnes* was routinely cultivated on Brain Heart Infusion Agar (BHIA), prepared by adding agar powder to BHIB. The culture was maintained under anaerobic conditions using a CO_2_ gas pack (Oxoid AnaeroGen 2.5L Sachet, Thermo Fisher Scientific Inc.) at 37°C for 48 h in an incubator (Jisico Co., Ltd., Republic of Korea).

**Disc diffusion assay.** The antimicrobial activity of the QsB extracts against *C. acnes* was assessed by the disc diffusion method [20]. Upon activation, the absorbance of *C. acnes* was adjusted to OD_600_ = 0.1 (equivalent to 1 × 10^6^ colony-forming units (CFU)/ml). Subsequently, 100 μl of the activated *C. acnes* culture was spread evenly onto BHIA plates using a cotton swab. Each QsB extract sample was then impregnated on sterilized 8 mm paper discs (ADVANTEC Toyo Roshi Kaisha., Ltd., Japan), which were placed at equal intervals on the BHIA plates. DMSO was used as the negative control. Following anaerobic incubation at 37°C for 48 h, the diameter of the resulting inhibition zones was measured.

**Minimum inhibitory concentration (MIC) assay.** To determine the antimicrobial activity of the QsB extracts, an MIC assay was performed using a two-fold serial dilution method. For the MIC assay, *C. acnes* was cultured overnight at 37°C in BHIB. Serial dilutions of the QsB extract (1‐1,000 μg/ml) were prepared, and DMSO served as the negative control. Then, 198 μl of the bacterial suspension was added to a 96-well plate (SPL Life Sciences Co., Ltd., Republic of Korea) containing 2 μl of QsB extract. After 24 h at 37°C, bacterial growth was visually assessed. The MIC was defined as the lowest concentration of QsB extract that suppressed visible microbial growth (no turbidity).

**Minimum bactericidal concentration (MBC) assay.** After establishing the MIC values, MBC assays were conducted as follows: 100 μl of bacterial suspension from the MIC wells showing no visible growth was spread onto fresh agar plates and evenly distributed. The plates were incubated anaerobically for 24 h at 37°C, and bacterial activity was evaluated by visual inspection.

**Biofilm inhibition assay.** To assess the potential of QsB extracts in inhibiting *C. acnes* biofilm formation, a modified crystal violet assay was conducted [3]. *C. acnes* was cultured on BHIA plates at 37°C for 48 h, and then adjusted to an OD_600_ of 2.0 in BHIB. Subsequently, 99 μl of the activated *C. acnes* suspension and 1 μl of QsB extract were added to the wells, with the control wells receiving only the solvent (DMSO). After 72 h of anaerobic incubation at 37°C, the supernatant was removed, and the wells were washed twice with 100 μl of sterile PBS. The remaining adherent cells (biofilm) were fixed with 100 μl of 99% methanol for 15 min before being air-dried. A 0.1% crystal violet solution was added for staining, followed by washing with tap water. After drying, 160 μl of 33% acetic acid was added to solubilize the crystal violet dye, and the solution was incubated for 20 min. Biofilm formation was quantified by measuring the OD_590_ using a microplate reader (Tecan Sunrise).

**Bacterial lipase inhibition assay.** A modified method was employed to determine the inhibition of *C. acnes* lipase enzyme activity [21]. *C. acnes* cells were ultrasonicated for 30 sec and immediately placed on ice to minimize enzyme degradation. After centrifugation at 12,000 × *g* for 3 min, the supernatant was collected as the crude enzyme. The reaction mixture contained 50 μl of the enzyme, 50 μl of the sample, and 100 μl of 0.1 mM 4-methylumbelliferyl oleate in buffer. The reaction proceeded for 10 min at 37°C under light. To terminate the reaction, 100 μl of 0.1 M sodium citrate solution (pH 4.2) was added. Fluorescence intensity was measured using a fluorescence plate reader (SpectraMax Gemini EM, Molecular Devices, USA) with excitation (Ex) at 355 nm and emission (Em) at 460 nm.

**Quantitative RT-PCR for gene expression analysis.** The gene expression in the fraction-treated *C. acnes* was assessed using quantitative real-time PCR (qRT-PCR). After treating *C. acnes* with various concentrations of the fractions (QsB-EE, E1, and E5) based on the MICs, the bacterial pellets were collected by centrifugation at 13,500 × *g* for 2 min (Vision Scientific Co.). Total RNA was extracted using a modified Trizol method [22]. RNA purity and concentration were measured with a spectrophotometer (Optizen-NanoQ, Mecasys Co., Ltd, Republic of Korea). Gene expression analysis was performed using the TOPreal SYBR Green RT-qPCR Kit (Enzynomics) and a qRT-PCR system (CFX Connect PCR System, Bio-Rad Laboratories, USA). Relative expression levels of the target genes were compared to the control group.

### Phytochemical Profiling and Compound Identification

**Fractionation by preparative HPLC.** The QsB-EE, which was obtained from the 80% methanol extract, was further separated by preparative HPLC to yield six subfractions (E1-E6) according to their elution order. Each subfraction was collected according to the elution order based on the major peaks observed in the chromatograms. Multiple preparative HPLC runs (LC- forte/R, YMC Co., Japan) were performed to separate the QsB-EE using a YMC-Triart Prep C18-S column (250 mm × 10.0 mm, i.d. 10 μm) (YMC Co.). Before the fractionation, the column was equilibrated at room temperature for 10 min. Then, QsB-EE was diluted to 400 mg/ml in HPLC-grade methanol, filtered through a 0.45 μm syringe filter, and then 400 μl was injected into the HPLC column. The mobile phase consisted of 0.1% formic acid dissolved in filtered HPLC-grade water (Solvent A) and HPLC-grade methanol (Solvent B). The gradient setting for the QsB-EE was as follows: 0-3 min: 20% B; 3-25 min: 20% → 80% B; 25-30 min: 80% B. The total run time was 30 min. Peak identification was performed with a diode array detector (DAD), and chromatograms were monitored at 220 nm, 280 nm, and 320 nm. Data acquisition and processing were conducted using the Clarity chromatography software (DataApex, the Czech Republic). The flow rate was 4.0 ml/min.

**LC-MS/MS analysis for compound identification.** The LC‐MS/MS analysis was performed using an Agilent 1290 Infinity II HPLC system (Agilent Technologies, Germany) coupled with a hybrid quadrupole time-of-flight (Q‐TOF) mass spectrometer (ZenoTOF 7600 MS, AB Sciex Pte. Ltd., USA). The mass spectrometer was operated in the negative ionization mode to detect [M‐H]− ions. Separation was conducted using a High-Strength Silica T3 (HSS T3) column (2.1 × 100 mm, 1.8 μm) (Waters, Republic of Korea). The electrospray ionization (ESI) method was conducted with the following settings: gas temperature 500°C, curtain gas 35 psi, ion source gas 1 (nebulizer gas) 50 psi, ion source gas 2 (heater gas) 50 psi, and spray voltage −4,500 V. The mobile phase comprised HPLC-grade water (Solvent A) and ACN (Solvent B).

**Statistical analysis.** All experiments were conducted in triplicate, and the collected data were statistically analyzed by one-way analysis of variance (ANOVA) using SPSS Statistics version 25.0 software (SPSS Inc., USA). The results were expressed as the mean ± SD. Differences between the means were assessed using Duncan’s multiple range test. Statistical significance was considered at a *p*-value of < 0.05.

## Results

### Extraction Yield

The extraction yields of QsB obtained using various solvents (80% methanol, n-hexane, ethyl acetate, n-butanol, and water) are presented in Table 1. The aqueous extract showed the highest yield (39.28%), followed by the n-butanol (34.57%) and 80% methanol extracts (25.67%). Notably, the ethyl acetate extract yielded 16.07%, while the n-hexane extract showed the lowest yield (3.94%). The yields varied significantly depending on the solvent polarity.

### Antioxidant Activity of QsB Extracts

The antioxidant capacity of QsB extracts was assessed using DPPH and ABTS radical scavenging assays (Table 2). Among the organic solvent extracts, the QsB-EE showed the highest DPPH (90.53%) and ABTS (87.23%) radical scavenging activity. Conversely, the n-hexane extract exhibited the lowest activity, with its ABTS activity being particularly low (7.03%). The 80% MeOH and n-butanol extracts exhibited the highest total phenolic (430.00 mg/g CAE) and total flavonoid contents (425.83 mg/g NE), respectively, followed by QsB-EE (230.83 mg/g CAE and 200.91 mg/g NE). The relatively high levels of phenolic and flavonoid compounds in QsB-EE were consistent with its potent DPPH (90.53%) and ABTS (87.23%) radical scavenging activities.

### Antimicrobial Activity against *C. acnes*

**Disc diffusion and minimum inhibitory/bactericidal concentration.** The antimicrobial activity of QsB-EE against *C. acnes* was evaluated using the paper disc diffusion method and broth microdilution to determine MIC/MBC values (Table 3). QsB-EE exhibited the largest inhibition zone compared to other tested extracts. Specifically, the inhibition zone for QsB-EE was 12.63 mm at a concentration of 0.5 mg/disc. Furthermore, MIC and MBC values were determined. QsB-EE showed an MIC of 125 μg/ml and an MBC of 250 μg/ml, confirming its potent inhibitory and bactericidal effects against *C. acnes*.

### Growth Inhibition Assay

**Fig. 1:**
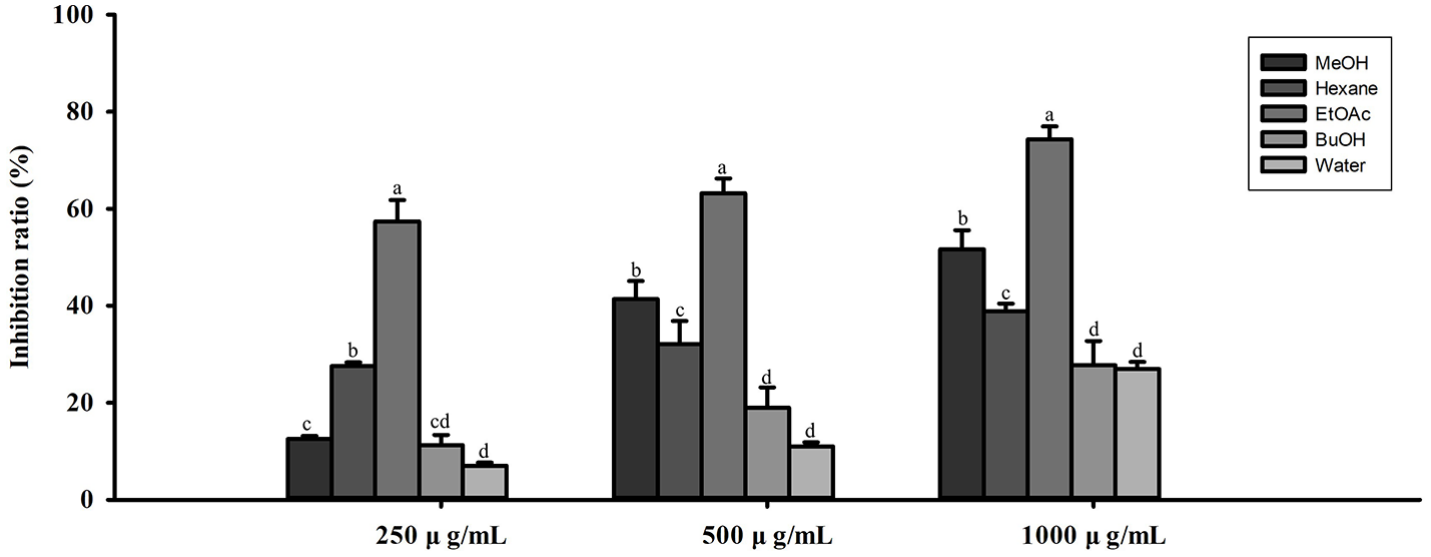
Inhibitory effects of *Quercus salicina* Blume extracts obtained with different solvents (MeOH, Hexane, EtOAc, BuOH, and Water) against *Cutibacterium acnes*. Values are expressed as mean ± SD (n = 3). Bars with different letters (a–d) indicate significant differences by one-way ANOVA and Duncan’s multiple range test (*p* < 0.05).

The growth inhibitory effects of different solvent extracts on *C. acnes* were further evaluated (Fig. 1). The 80% methanol extract (41.37% at 500 μg/ml) and the n-hexane extract (38.83% at 1,000 μg/ml) exhibited significant inhibitory effects. In contrast, the n-butanol and water extracts showed only limited inhibitory activity (approximately 27-28% at maximum concentration). Consistently, QsB-EE demonstrated the most potent inhibitory activity, reaching 74.32% inhibition at 1,000 μg/ml and showing strong inhibition even at 250 μg/ml (57.38%), further highlighting its superior efficacy against *C. acnes*.

### Fractionation of QsB-EE and Antimicrobial Evaluation

**Fig. 2:**
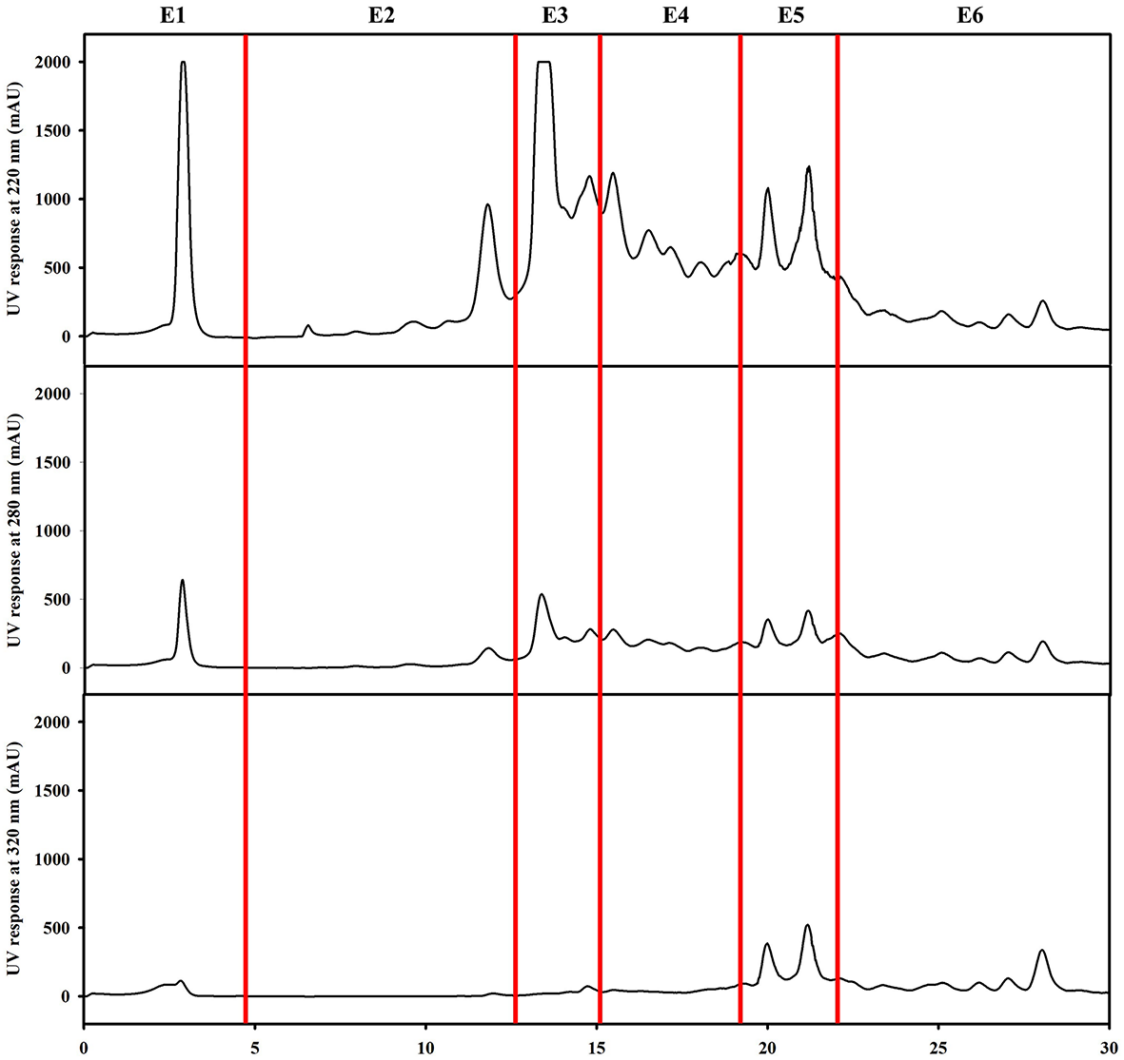
Preparative HPLC chromatograms of the ethyl acetate fraction (QsB-EE) of *Quercus salicina* Blume. The chromatograms were monitored at 220, 280, and 320 nm using a YMC-Triart Prep C18-S column (250 × 10 mm, 10 μm) at a flow rate of 4.0 ml/min. Fractions E1–E6 were collected according to the major peaks observed under a linear gradient of 20–80% methanol containing 0.1% formic acid over 30 min.

**Fig. 3:**
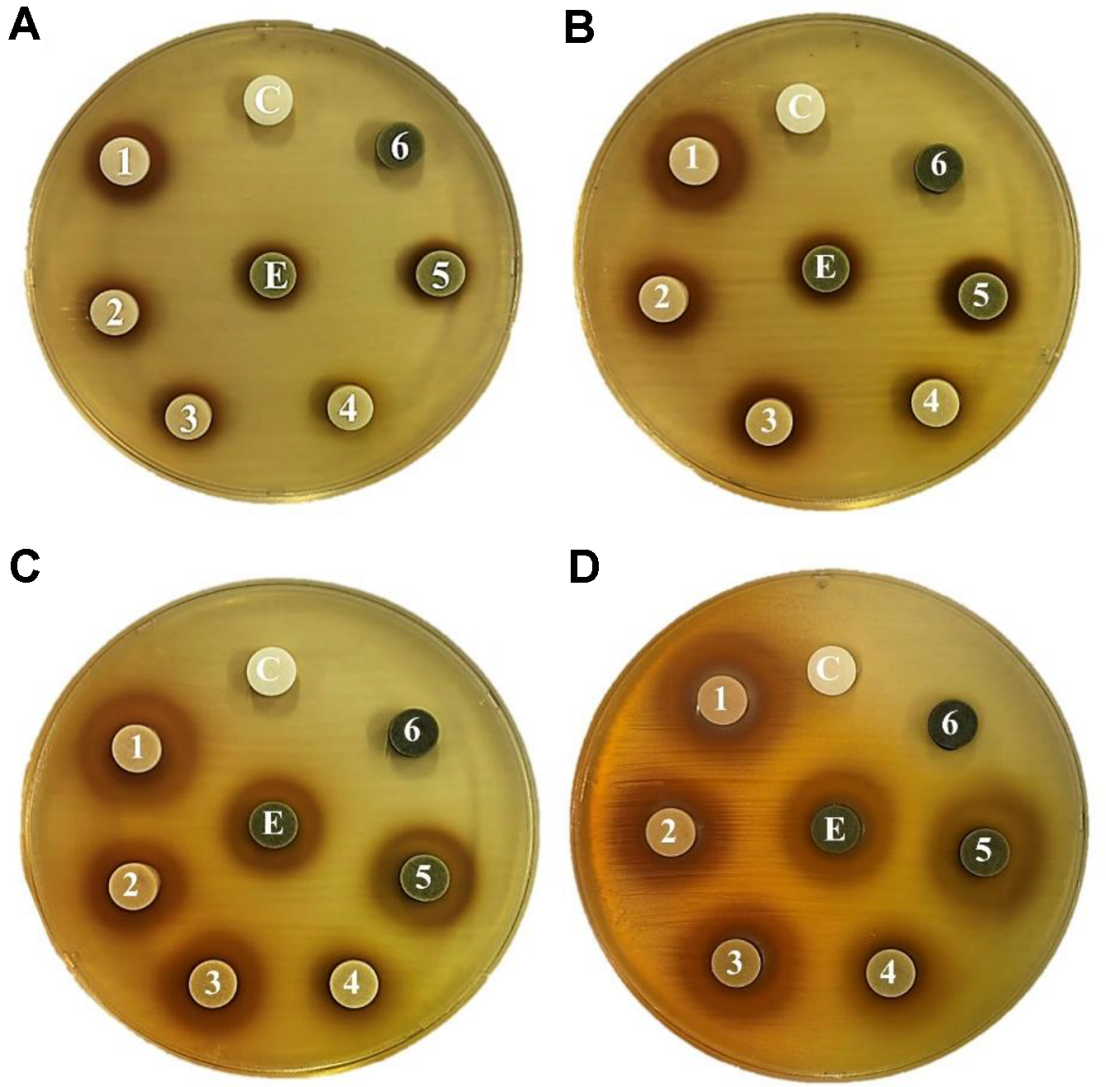
Antibacterial activity of *Quercus salicina* Blume ethyl acetate extract (QsB-EE) and its subfractions (E1–E6) against *Cutibacterium acnes* by the disc diffusion method. Each disc contained 0.5 mg (**A**), 1 mg (**B**), 2 mg (**C**), or 5 mg (**D**) of the sample. E = QsB-EE; 1–6 = subfractions; C = DMSO (negative control). Plates were incubated anaerobically at 37°C for 48 h.

QsB-EE was fractionated into six subfractions (E1 to E6) by preparative HPLC, based on their elution profile (Fig. 2). The antimicrobial activities of these fractions were evaluated using disc diffusion assays, MIC, and MBC (Fig. 3, Table 4).

Fractions E1 and E5 exhibited the strongest antimicrobial activity, with their inhibition zones surpassing that of the unfractionated QsB-EE. This enhanced efficacy was confirmed by the MIC and MBC assays. Both fractions demonstrated the lowest MIC value (16 μg/ml) among all subfractions. The corresponding MBC values were determined to be 125 μg/ml for E1 and 250 μg/ml for E5, highlighting the selective concentration of antimicrobial compounds within these two fractions.

### Inhibition of Biofilm Formation

**Fig. 4:**
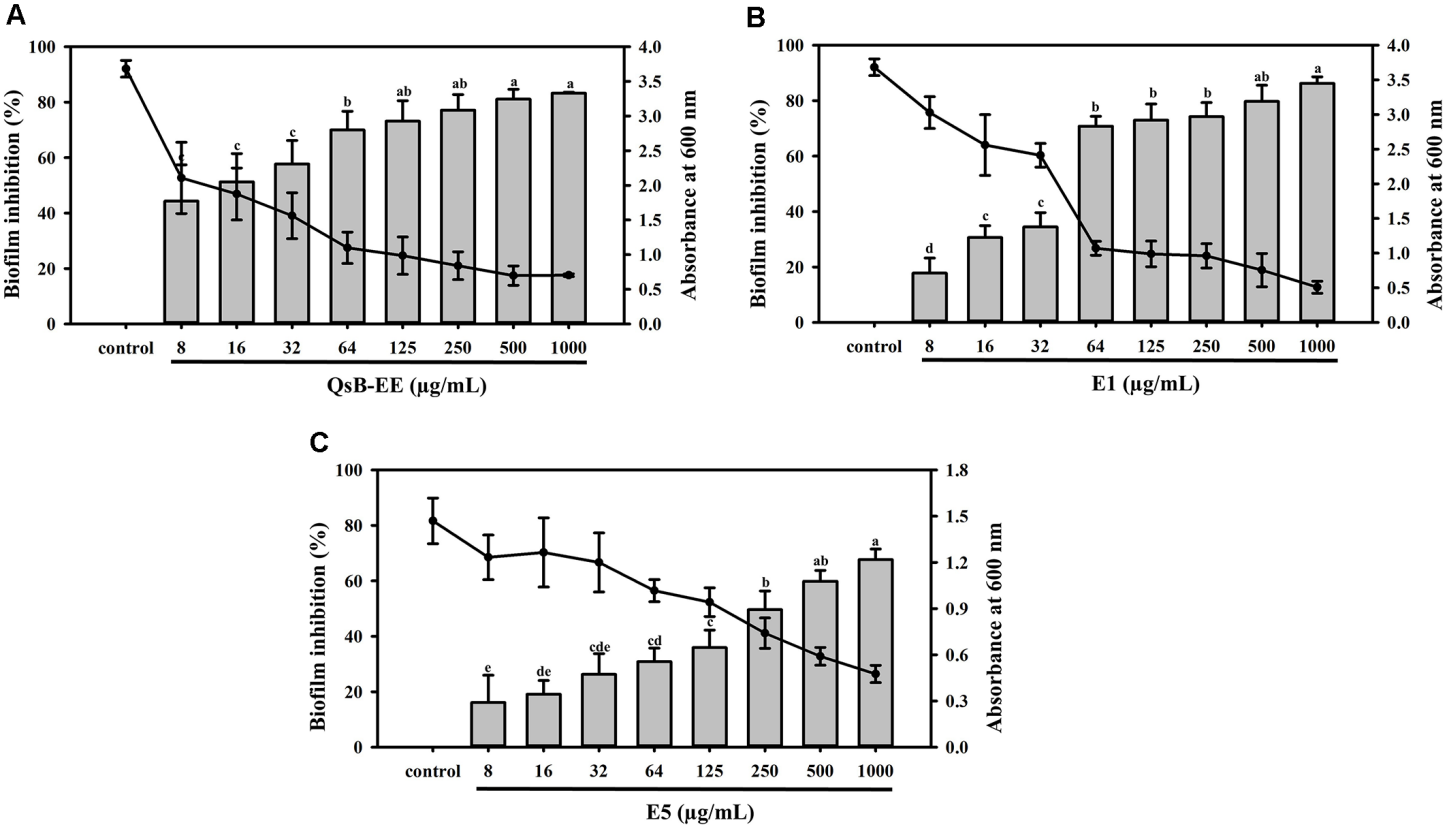
Antibiofilm effects of *Quercus salicina* Blume extracts and fractions against *Cutibacterium acnes*. (**A**) QsB-EE, (**B**) E1, and (**C**) E5. Biofilm inhibition (%) and absorbance at 590 nm represent the percentage of biofilm reduction and residual biomass, respectively. Values are expressed as mean ± SD (n = 3). Bars with different letters (a–e) indicate significant differences according to one-way ANOVA and Duncan’s multiple range test (*p* < 0.05).

The antibiofilm activities of QsB-EE and its potent fractions E1 and E5 are presented in Fig. 4. At a concentration of 1,000 μg/ml, the fractions and extract showed strong inhibitory effects on *C. acnes* biofilm formation. Fraction E1 demonstrated the highest inhibition (86.19%), followed by the unfractionated QsB-EE (82.24%) and fraction E5 (67.64%). Notably, E5's biofilm inhibition was less pronounced than that of E1 and QsB-EE, suggesting that E1 is specifically enriched with compounds responsible for anti-biofilm activity, independent of the general bactericidal effect.

### Inhibition of Bacterial Lipase Activity

**Fig. 5:**
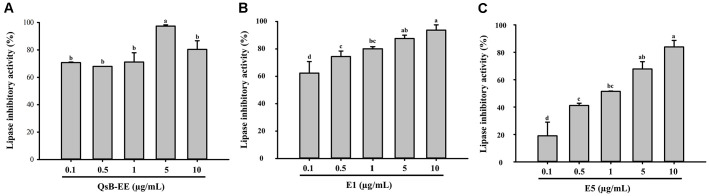
Lipase inhibitory activity of *Quercus salicina* Blume extracts and fractions against *Cutibacterium acnes*. (**A**) QsB-EE, (**B**) E1, and (**C**) E5. Lipase inhibition (%) was determined using a fluorescence-based assay with excitation at 355 nm and emission at 460 nm. Values are expressed as mean ± SD (n = 3). Bars with different letters (a–d) indicate significant differences according to one-way ANOVA and Duncan’s multiple range test (*p* < 0.05).

The lipase inhibitory activities of QsB-EE and its potent fractions E1 and E5 are presented in Fig. 5. At a concentration of 10 μg/ml, Fraction E1 demonstrated the highest inhibition (93.72%) of *C. acnes* lipase activity. Fraction E5 (83.87%) and the unfractionated QsB-EE (81.23%) also showed strong inhibitory effects, indicating that the active compounds responsible for lipase inhibition are highly concentrated in the E1 fraction.

### Downregulation of *C. acnes* Virulence Genes

**Fig. 6:**
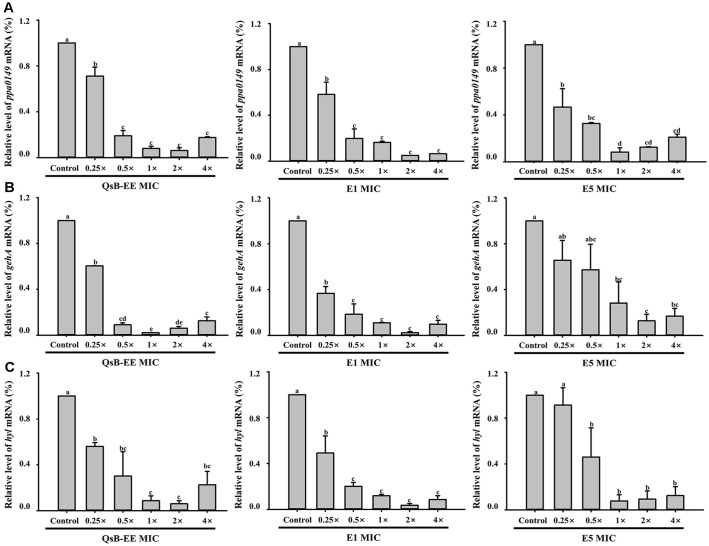
Effects of *Quercus salicina* Blume EtOAc extract (QsB-EE), fraction E1, and fraction E5 on the mRNA expression of *Cutibacterium acnes* virulence-related genes. (A) *ppa*0149, (B) *geh*A, and (C) *hyl*. The relative expression levels were determined by qRT-PCR using the 16S rRNA gene as the internal control. Values are expressed as mean ± SD (n = 3). Bars with different letters (a–e) indicate significant differences according to one-way ANOVA and Duncan’s multiple range test (*p* < 0.05).

qRT-PCR was performed to evaluate the effects of QsB-EE, E1, and E5 on the expression of *C. acnes* virulence-associated genes (Fig. 6). Treatment with these fractions resulted in the significant downregulation of the genes *ppa*0149 (biofilm formation), *gehA* (lipase activity), and *hyl* (hyaluronate lyase).

As shown in Fig. 6A, *ppa*0149 expression was markedly reduced, with E1 treatment showing the greatest inhibitory effect, particularly at lower concentrations. This finding is consistent with the results of the biofilm inhibition assay. Similarly, *gehA* expression, associated with lipase activity, was significantly downregulated by all fractions, with E1 again exhibiting the greatest inhibitory effect (Fig. 6B). Consistent with its known link to tissue degradation, *hyl* expression was also substantially reduced by QsB-EE, E1, and E5, even at lower concentrations (Fig. 6C).

### Identification of Bioactive Compounds in the QsB-EE Fractions

**Fig. 7:**
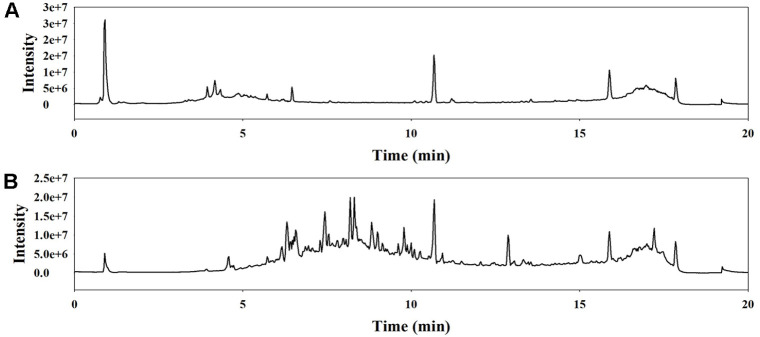
Total ion chromatograms (TICs) of *Quercus salicina* Blume fractions obtained by LC–MS/MS analysis. (**A**) E1 and (**B**) E5 fractions. Chromatographic separation was performed on a High Strength Silica T3 (2.1 × 100 mm, 1.8 μm) column using water (Solvent A) and acetonitrile (Solvent B) as the mobile phase. Detection was carried out in negative electrospray ionization (ESI) mode with a spray voltage of -4,500 V. Peaks represent detected metabolites according to their retention time and relative intensity.

LC-MS/MS analysis in the ESI negative ionization mode was performed to identify the key antimicrobial compounds in fractions E1 and E5. The total ion chromatograms for these fractions are presented in Fig. 7, and the identified compounds are summarized in Table 5.

**Fig. 8:**
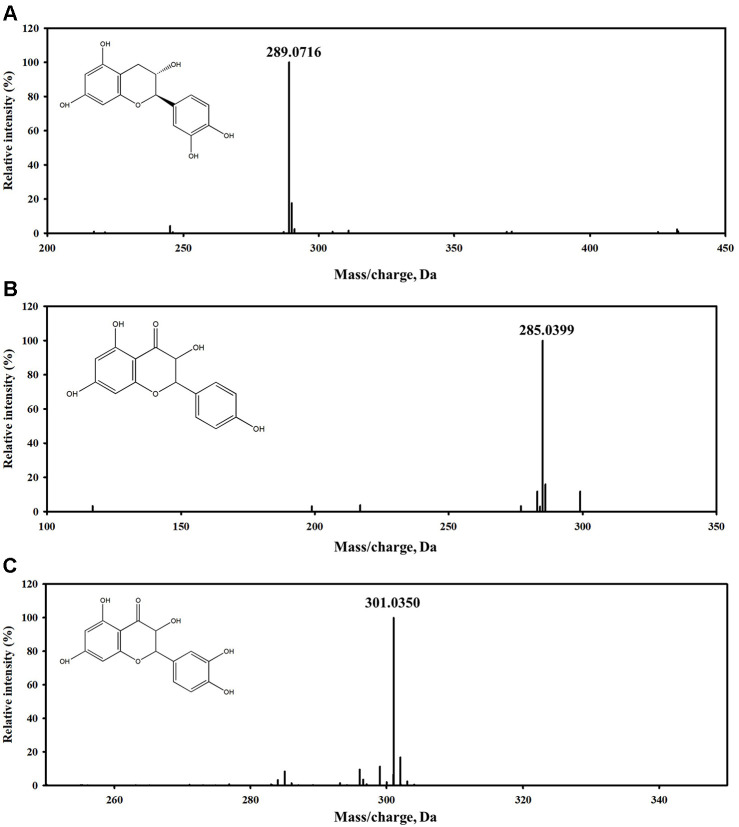
LC–MS/MS spectra and chemical structures of major compounds identified in *Quercus salicina* Blume fractions. (**A**) (+)-Catechin in E1 ([M−H]^-^, *m/z* ≈289.07), (**B**) kaempferol in E5 ([M−H]^-^, *m/z* ≈285.04), and (**C**) quercetin in E5 ([M−H]^-^, *m/z* ≈301.04).

In Fraction E1, the primary compound identified was (+)-catechin ([M−H]^−^, *m/z* 289.072) (Fig. 8A), a compound recognized for its strong antioxidant and antimicrobial properties. In Fraction E5, kaempferol ([M−H]^−^, *m/z* 285.040) and quercetin ([M−H]^−^, *m/z* 301.035) were identified as the major constituents (Fig. 8B and 8C). The identification of these known bioactive compounds, which are recognized for their potent antioxidant, anti-inflammatory, and antimicrobial activities, supports the observed efficacies of the QsB-EE fractions.

To ensure accurate annotation, compound identification was conducted by matching the acquired MS/MS spectra with the Sciex LibraryView spectral library integrated into the instrument software. Library matching was performed using the standard LibraryView dot-product–based scoring algorithm. To further increase annotation confidence, the acquired spectra were cross-validated using MassBank.eu (MassBank of Europe), which provides experimentally validated reference MS/MS spectra for flavonoids and other plant-derived compounds. Diagnostic fragment ions for flavonoids were additionally compared against previously reported fragmentation data in the literature. Because authentic standards were not analyzed in parallel, all compounds were classified as Level 2 (putatively identified based on MS/MS spectral similarity) according to widely accepted metabolite identification guidelines, corresponding to the Metabolomics Standards Initiative (MSI) Level 2 definition.

## Discussion

The extraction yield was inversely correlated with solvent hydrophobicity, suggesting that polarity significantly influences the extraction efficiency of bioactive compounds. The low phenolic content in the n-hexane extract suggests that non-polar solvents predominantly extract lipophilic compounds (*e.g.*, fats and waxes), which may exhibit weaker radical scavenging activity than phenolic compounds. Despite yielding the highest amount of plant material, the water extracts did not exhibit high antimicrobial efficacy, emphasizing that extraction yield does not necessarily correlate with antimicrobial activity. This observation aligns with earlier studies indicating that organic solvents, such as acetone and ethyl acetate, are more effective in extracting antimicrobial compounds compared to water-based solvents [23].

The antioxidant activity observed in the QsB-EE can be attributed to its high flavonoid and phenolic content. Similar results were reported in *Quercus incana* Roxb. extracts, where the highest DPPH radical scavenging activity was observed in the butanol and ethyl acetate extracts [24]. These findings are consistent with the notion that phenolic compounds possess antioxidant activity due to their structural ability to donate electrons or hydrogen atoms [25]. Furthermore, phenolic compounds and flavonoids are known to exhibit various beneficial properties, including antimicrobial, anticancer, anti-inflammatory, and antioxidant effects [26-28]. Oxidative stress is known to contribute to acne pathophysiology, where processes such as squalene peroxidation and ROS accumulation can promote follicular inflammation and facilitate *C. acnes*–induced activation of pro-inflammatory cytokines [29]. In this context, the strong antioxidant capacity and high phenolic and flavonoid content observed in QsB-EE may help modulate oxidative and inflammatory processes associated with acne development. Moreover, catechin, quercetin, and kaempferol—identified in the active fractions—have been reported to modulate ROS levels and inflammatory signaling in various skin-relevant experimental models, providing supportive mechanistic relevance for their potential involvement in acne-related pathways [30].

Antimicrobial assays revealed that QsB-EE exhibited potent activity against *C. acnes*, with fractions E1 and E5 showing the most significant effects. Notably, E1 showed superior overall activity in the growth inhibition assay and stronger antibiofilm activity than E5. This suggests that, while both fractions are effective in suppressing bacterial proliferation (MIC 16 μg/ml), E5's impact on biofilm-associated mechanisms may be limited compared to E1. In a previous study, *Quercus calliprinos* extracts did not demonstrate clear zones against *C. acnes* at 1.25 mg/disc [31]. Antimicrobials are typically classified as bactericidal if the MBC/MIC ratio is ≤ 4, and as bacteriostatic if the ratio exceeds 4 [32]. For all the tested QsB extract samples, the ratios obtained were under 4, indicating that all the samples exhibited significant bactericidal activity against *C. acnes*.

Biofilm formation, a pivotal step in disease progression, hinges significantly on the initial adherence of microorganisms to surfaces [3]. Bacterial adherence to surfaces, including those of polystyrene plates, is influenced by factors such as charge, hydrophobicity, and the production of extracellular polysaccharides [33]. The ability of biofilms to enhance keratinocyte compaction may exacerbate follicular blockage and inflammation, contributing to acne severity [34]. The strong antibiofilm activity of E1 suggests its potential to disrupt this critical initial adherence and subsequent keratinocyte compaction, thereby mitigating acne progression.

The inhibition of *C. acnes* lipase is crucial, as the free fatty acids released from triglyceride hydrolysis promote ductal hypercornification and enhance bacterial adhesion to keratinocytes and follicle cells [35]. This adhesion facilitates *C. acnes* colonization and biofilm formation, exacerbating acne pathogenesis [36]. The use of QsB-EE and its fractions would significantly prevent sebum degradation by *C. acnes*, potentially reducing the release of free fatty acids and the formation of glycerol as a nutrient source.

The qRT-PCR analysis provided further insights into the antimicrobial mechanisms of QsB-EE and its fractions. The expression of all virulence-associated genes decreased with increasing extract concentrations. Notably, fractions E1 and E5 were more effective than the unfractionated QsB-EE in suppressing both bacterial growth and virulence factor expression. These findings provide evidence of the potential of QsB-EE and its fractions to effectively modulate the gene expression of *C. acnes* relevant to acne vulgaris.

The identification of (+)-catechin in the E1 fraction suggests its contribution to the observed antimicrobial and antioxidant activities, consistent with previous studies highlighting its antidiabetic, antimicrobial, and anti-inflammatory properties [37, 38]. The distinct composition of E1 (primarily (+)-catechin) compared to E5 (kaempferol and quercetin) may account for the superior anti-biofilm and gene-modulating effects observed in E1. Both kaempferol and (+)-catechin are potential candidates for the treatment of acne vulgaris due to their significant inhibitory activity on *C. acnes* lipase *gehA* [39]. Kaempferol has demonstrated anticancer, antioxidant, and anti-inflammatory activities, and shows antimicrobial activity against *Porphyromonas gingivalis*, *Prevotella intermedia*, and *C. acnes* [40-44]. Quercetin, named after the genus *Quercus*, has been widely studied for its anti-inflammatory, antioxidant, antibiofilm, and antimicrobial properties, making it a promising candidate for health applications [45-48]. The major polyphenolic constituents identified in the QsB-EE—catechin, kaempferol, and quercetin—are known to possess intrinsic antimicrobial and antioxidant activities. Crucially, the overall efficacy of the complex extract was greater than that expected from the sum of its single components, suggesting a synergistic or multi-target contribution among its active molecules. While quantitative verification of this synergy was beyond the scope of the current study, the potent activity observed warrants further investigation into the potential complementary roles of these co-existing compounds. Future studies should therefore employ purified compounds to quantitatively validate these synergistic interactions.

To ensure safety for topical application, rigorous management of residual solvents used in the extraction process (methanol, n-hexane, ethyl acetate, and n-butanol) was paramount. The solvent removal relied on a two-step validated purification process: rotary evaporation for the efficient bulk removal of volatile solvents, followed by lyophilization (freeze-drying) under high vacuum to eliminate trace amounts of residual, less volatile components, such as n-butanol. This dual approach ensures that any remaining organic solvent is reduced to negligible levels, well within acceptable safety limits for dermal products.

The safety profile is further supported by the traditional use of the *Q. salicina* species and the known favorable dermal tolerability of its key constituents (catechin, kaempferol, and quercetin). When formulated at low concentrations (typically ≤ 1% w/w), the potential exposure to any trace residue remains below toxicologically relevant thresholds.

Although the extract was prepared using an organic solvent, the present study did not include toxicological assessments, such as residual solvent quantification, cytotoxicity assays, or patch testing. Therefore, no definitive conclusions regarding its safety profile can be drawn at this stage. Future studies should incorporate GC-based residual solvent analysis, *in vitro* cytotoxicity testing on relevant skin cell models (*e.g.*, keratinocytes), and human patch testing to establish a quantitative margin of safety for potential topical applications.

## Conclusion

QsB-EE, particularly fractions E1 and E5, exhibited strong antimicrobial and anti-virulence effects against *C. acnes*, inhibiting bacterial growth, biofilm formation, and lipase activity. This effect was further substantiated by mechanistic analysis, which revealed that QsB-EE downregulated key virulence genes (*ppa*0149, *geh*A, and hyl), disrupting *C. acnes* pathogenicity. LC-MS/MS analysis identified catechin, kaempferol, and quercetin as the major bioactive compounds contributing to these effects. These findings suggest that QsB-EE is a promising natural alternative for acne treatment, warranting further *in vivo* and clinical validation.

## Figures and Tables

**Table 1:** Extraction yields (%) of organic solvent extracts from *Quercus salicina* Blume.

Extracts	Extraction yields (%)
80% methanol	25.67 ± 0.40
n-hexane	3.94 ± 1.89^d^
Ethyl acetate	16.07 ± 0.41^c^
n-butanol	34.57 ± 1.51^b^
Water	39.28 ± 0.56^a^

Values are expressed as mean ± SD (n = 3). Different letters (a–d) indicate significant differences among groups as determined by Duncan’s multiple range test (*p* < 0.05).

**Table 2:** Antioxidant activities and compositional indices of *Quercus salicina* Blume extracts.

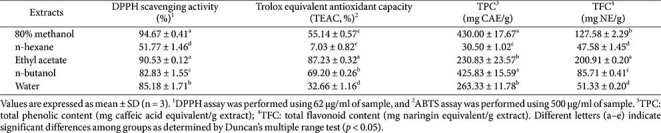

**Table 3:** Antimicrobial activity (disc diffusion, MIC, and MBC) of *Quercus salicina* Blume extracts against *Cutibacterium acnes*.

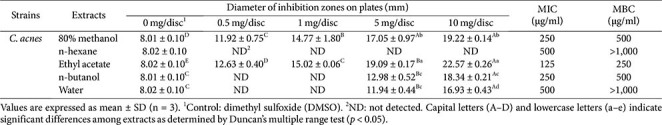

**Table 4:** Antimicrobial activity (disc diffusion, MIC, and MBC) of *Quercus salicina* Blume fractions (E1–E6) derived from the EtOAc extract (QsB-EE) against *Cutibacterium acnes*.

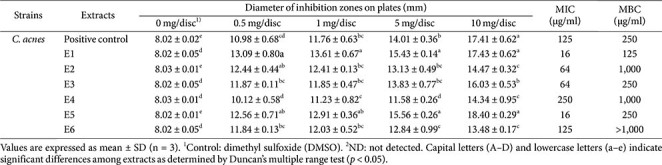

**Table 5:** Identified compounds in fraction E1 and E5 of *Quercus salicina* Blume by LC–TOF–MS/MS analysis.